# Breaking Down Osteoarthritis: Exploring Inflammatory and Mechanical Signaling Pathways

**DOI:** 10.3390/life15081238

**Published:** 2025-08-04

**Authors:** Wafa Ali Batarfi, Mohd Heikal Mohd Yunus, Adila A. Hamid, Manira Maarof, Rizal Abdul Rani

**Affiliations:** 1 Department of Physiology, Faculty of Medicine, Universiti Kebangsaan Malaysia, Jalan Yaacob Latiff, Bandar Tun Razak, Kuala Lumpur 56000, Malaysia; w.batarfi@hu.edu.ye (W.A.B.); adilaha-mid@ppukm.ukm.edu.my (A.A.H.); 2 Department of Basic Medical Sciences, Hadhramout University College of Medicine, Al-Mukalla, P.O. Box 8892, Yemen; 3 Department of Tissue Engineering and Regenerative Medicine, Faculty of Medicine, Universiti Kebangsaan Malaysia, Jalan Yaacob Latiff, Bandar Tun Razak, Kuala Lumpur 56000, Malaysia; manira@ppukm.ukm.edu.my; 4 Department of Orthopaedics and Traumatology, Faculty of Medicine, Universiti Kebangsaan Malaysia, Jalan Yaacob Latiff, Bandar Tun Razak, Kuala Lumpur 56000, Malaysia; rizalabdulrani@gmail.com

**Keywords:** osteoarthritis, chondrocytes, inflammation, mechanical signaling, cytokines, cartilage degradation

## Abstract

Osteoarthritis (OA) is a chronic progressive joint disease characterized by cartilage degradation, subchondral bone remodeling, and synovial inflammation. This complex disorder arises from the interplay between mechanical stress and inflammatory processes, which is mediated by interconnected molecular signaling pathways. This review explores the dual roles of inflammatory and mechanical signaling in OA pathogenesis, focusing on crucial pathways such as NF-kB, JAK/STAT, and MAPK in inflammation, as well as Wnt/β-catenin, Integrin-FAK, and Hippo-YAP/TAZ in mechanotransduction. The interplay between these pathways highlights a vicious cycle wherein mechanical stress exacerbates inflammation, and inflammation weakens cartilage, increasing its vulnerability to mechanical damage. Additionally, we discuss emerging therapeutic strategies targeting these pathways, including inhibitors of cartilage-degrading enzymes, anti-inflammatory biologics, cell-based regenerative approaches, and non-pharmacological mechanical interventions. By dissecting the molecular mechanisms underlying OA, this review aims to provide insights into novel interventions that address both inflammatory and mechanical components of the disease, paving the way for precision medicine in OA management.

## 1. Introduction

OA is a chronic, progressive, degenerative joint condition characterized by articular cartilage destruction and subchondral bone abnormalities [1]. It is a primary cause of disability, globally affecting over 240 million people worldwide. A comprehensive analysis of 88 studies, involving over 10 million participants, revealed that the global prevalence of OA is 16.0% in adults aged 15 and older, rising significantly to 22.9% in those aged 40 and above. The study further indicated that OA prevalence was 1.69 times higher in females compared to males, with an incidence also elevated in females, resulting in an incidence ratio of 1.39%. The occurrence of OA additionally showed a positive correlation with age, peaking among those aged 70 to 79 years. This steady rise in OA prevalence worldwide is largely explained by the aging population and the obesity epidemic [2,3,4,5]. The socioeconomic costs of OA are significant, in addition to the impact it has on individuals; as a result, OA constitutes a major health issue for the ensuing decades [6,7].

OA is associated with aging and repetitive mechanical pressures. Three primary subgroups of etiological factors have been identified by recent studies: body mass, anatomy, and sex. Clinical signs include pain and stiffness in the joints, reduced range of motion, quadriceps muscle weakness, and changes in proprioception [8]. Radiological changes include joint space narrowing, osteophyte formation, periarticular cysts, and subchondral sclerosis [9]. Reduced power in the muscles around the joints is important because it leads to a gradual loss of function. These symptoms severely limit the individual’s ability to move, climb stairs, and get out of a chair. Individuals with OA may also exhibit instability, poor limb alignment, and limping. Because of the uneven joint surfaces caused by arthritis, noises of crepitating might be heard during motions [10].

OA can be divided into two categories: primary and secondary OA. Idiopathic OA, another name for primary OA, is the term used to describe the degenerative changes in joints brought on by hereditary disorders without any established underlying causes. One may additionally categorize primary OA into monoarticular OA, which affects one joint, and polyarticular OA, which affects three or more joints. Injury or risk factors have been linked to secondary OA. A few conditions that are connected to secondary osteoarthritis include obesity, diabetes, food, physical activity, rheumatoid arthritis, and other disorders affecting the metabolism or bones [11,12].

To date, there is no effective or cure treatment for OA, only therapies that slow or prevent OA progression [13]. Although the only methods available for treating individuals with end-stage OA are still osteotomies and arthroplasties. Unfortunately, this method is invasive, and joint function will not be completely regained [14]. Therefore, understanding the dual roles of inflammation and mechanical stress in OA is fundamental for developing comprehensive therapeutic strategies aimed at both modulating inflammation and restoring joint biomechanics with tissue integrity [15].

This review attempts to clarify the complicated interplay between the mechanical and inflammatory signaling pathways and their roles in the progression of OA by methodically analyzing the present literature. Furthermore, the study aims to find possible therapeutic targets inside these pathways that can present novel directions for the management and therapy of OA.

This review is structured as follows: we begin by unraveling the pathophysiology of OA, which describes the core mechanisms that drive the illness. This is followed by a detailed review of the inflammatory response in OA and the distinctive inflammatory signaling pathways in OA. Concurrently, we are investigating the crucial role of mechanical stress in OA, as well as the underlying mechanotransduction signaling pathways. We then dedicate a section to investigating the critical interplay between inflammatory and mechanical signaling, emphasizing their synergistic contributions to disease progression. Finally, we cover emerging therapeutic strategies targeting inflammatory and mechanical pathways in OA, which include novel techniques to alter the disease course and improve patient outcomes.

## 2. Unravelling the Pathophysiology of OA

The pathophysiology of OA is complicated and lacks a comprehensive understanding [16]. Typically, healthy articular cartilage is composed of chondrocytes and extracellular matrix (ECM), including mainly type II collagen, proteoglycans, and water, which collectively contribute to its ability to absorb shocks [17]. Proteoglycan is composed of aggrecan and hyaluronan, which interact to create glycosaminoglycan (GAG). Preserving chondrocytes in cartilage is crucial for maintaining joint health due to the absence of blood vessels or nerves in articular cartilage and its limited ability for self-repair [18]. Chondrocytes regulate the homeostasis of the cartilage by producing ECM, which helps preserve the structure and function of the cartilage [19,20].

OA arises from the failure of chondrocytes to maintain a balance between the production and breakdown of the ECM [21]. During these instances, the immune cells will phagocytose the wear particles generated in the joints, leading to elevated production and activation of lysozymes in response to the injury. If the level at which wear particles are created exceeds the level at which they are removed from the system, they will trigger an inflammatory reaction. This, in turn, will cause chondrocytes to release enzymes that break down collagen and proteoglycan in the joint [2,22].

Numerous immune-related and inflammatory cells, such as megakaryocytes, neutrophils, macrophages, lymphocytes, leukocytes, and dendritic cells, are produced as a natural defense in the immune response [23,24]. A complex network of components, including proinflammatory cytokines, chemokines, and lipid mediators, binds to chondrocytes and coordinates immune cell behaviors by triggering signal transduction pathways [19]. Consequently, an increased number of metalloproteinases will be produced while the synthesis of type II collagen is suppressed. As a result, the breakdown of cartilage accelerates, leading to an increase in the apoptosis of chondrocytes.

**Figure 1:**
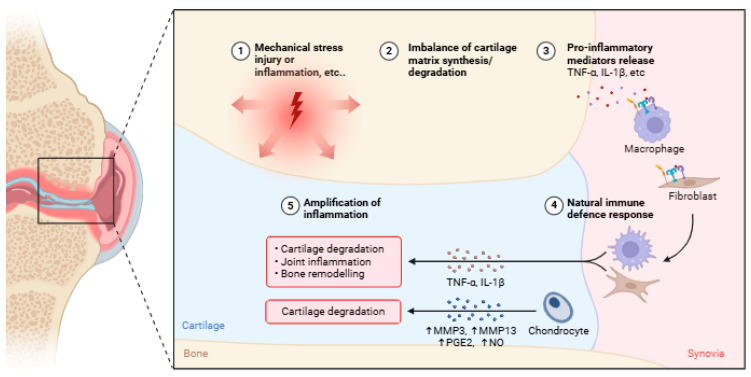
Pathogenesis of OA begins with (1) an initial trigger such as mechanical stress, injury, or inflammation (arrows indicate that mechanical stress, injury, or inflammation are primary triggers that set off the destructive cascade within the joint), leading to (2) an imbalance where cartilage degradation outpaces synthesis. This process involves (3) release of pro-inflammatory mediators (e.g., TNF-α, IL-1β) and (4) a natural immune defense response. Ultimately, a vicious feedback loop results in (5) amplification of inflammation, exacerbating joint damage, cartilage degradation, and bone remodeling. TNF-α: tumor necrosis factor-alpha; IL-1β: interleukin 1 beta; MMP: matrix metalloproteinase; PGE2: prostaglandin E2; NO: nitric oxide. Created with BioRender. (https://BioRender.com (accessed on 15 February 2025).

In contrast, several cytokines and chemokines have a significant impact on the development of OA. These include pro-inflammatory cytokines such as interleukin-1β (IL-1β), tumor necrosis factor-alpha (TNF-α), IL-6, IL-15, IL-17, and IL-18 [25]. IL-1β, TNF-α, and IL-6 are the primary inflammatory mediators in the development of OA. They initiate many signaling pathways that subsequently activate additional cytokines and pathological processes [14]. This inflammatory response is often amplified or initiated by mechanical overload, highlighting its synergistic contribution to OA progression. Additionally, cytokines can induce the synthesis of chemokines, which in turn attract additional inflammatory cells to the joint. This leads to an increased secretion of inflammatory substances and increases OA progression (Figure 1) [15].

## 3. Inflammatory Response in OA

Inflammation is regarded as a fundamental mechanism that is potentially linked to the pathophysiology of OA [26]. It is now well-established that chronic inflammation contributes to the progression of OA and is a key driver of cartilage degeneration in the joints [27]. Multiple factors, such as mechanical stress, joint damage, or improper biomechanics, might trigger this inflammatory response. Pro-inflammatory cytokines and chemokines, like IL-1β and TNF-α, are increased in osteoarthritic joints [25,28].

The cytokines function as autocrine and paracrine mediators to induce the joint synthesis of proteases, nitric oxide (NO), and eicosanoids such as prostaglandins (PE2) and leukotrienes by chondrocytes and macrophages [20]. The inflammatory mediators work on the cartilage to induce catabolic pathways, limit matrix production, and promote cellular apoptosis. Pro-inflammatory cytokines impede autophagy, leading to cellular apoptosis, especially in chondrocytes [29,30].

Inflammatory mediators, especially IL-1β, stimulate the production of matrix-degrading enzymes such as MMPs, mainly MMP1, MMP3, and MMP13 and ADAMTS [31]. These catabolic biomarkers hinder the production of essential components of the extracellular matrix in cartilage, such as proteoglycans, aggrecan, and type II collagen, causing its degradation, the loss of its structural integrity, and chondrocyte apoptosis [32].

Furthermore, fibronectin has a role in cartilage breakdown by stimulating chondrocytes to produce more inflammatory cytokines, chemokines, and MMPs when protein fragments are present [33,34]. Chondrocytes in typical mature cartilage usually produce matrix components at a low rate. Chondrocyte senescence significantly contributes to the development and progression of osteoarthritis. Senescent cells lose their ability to maintain and repair the cartilage ECM. IL-6 and IL-8 are important cytokines and chemokines that are released by senescent cells, known as the senescence-associated secretory phenotype [12,20,35].

In addition to proteases and cytokines, the levels of enzymes such as inducible NO synthase (iNOS) and cyclooxygenase-2 (COX-2) are changed in OA. iNOS produces the free radical NO, while COX-2 generates PGE2 [36]. IL-1β induces the gene expression or activity of COX-2 and iNOS, leading to the elevation of PGE2 and NO [37].

IL-1, in conjunction with mechanical loading of the cartilage, stimulated the upregulation of the iNOS gene, leading to an increase in NO generation. NO promotes articular disintegration by increasing MMPs synthesis through cGMP-dependent pathways and decreasing the production of proteoglycans and collagen [29,38]. NO is involved in regulating chondrocyte apoptosis, which is a typical characteristic in advanced OA. Furthermore, NO also changes the way mitochondria work in osteoarthritis chondrocytes, leading to decreased cell viability by blocking the function of the mitochondrial respiratory chain and ATP production [39,40].

## 4. Inflammatory Signaling Pathways in OA

The pathophysiology of OA is significantly influenced by inflammation, with multiple important mechanisms causing pain, cartilage deterioration, and synovial inflammation. Among the main inflammatory pathways are the following:

### 4.1. NF-kB Signaling Pathway

Many proteins and signaling pathways are involved in the control of the regulation of inflammation [41]. One of these inflammatory signaling pathways is the nuclear factor kappa-light-chain-enhancer of activated B cells (NF-kB), which is stimulated by different types of chemokines and inflammatory cytokines such as IL-6, IL-1β, and TNFα [42]. NF-kB is a group of transcription factors that has a central role in inflammation, cellular differentiation, proliferation, and survival of normal and malignant cells [43,44]. Attributable to the wide-ranging biological involvement of NF-kB, dysregulation of NF-kB pathways is often seen in a variety of disorders, including cancer, autoimmune diseases, and arthritis [45].

**Figure 2:**
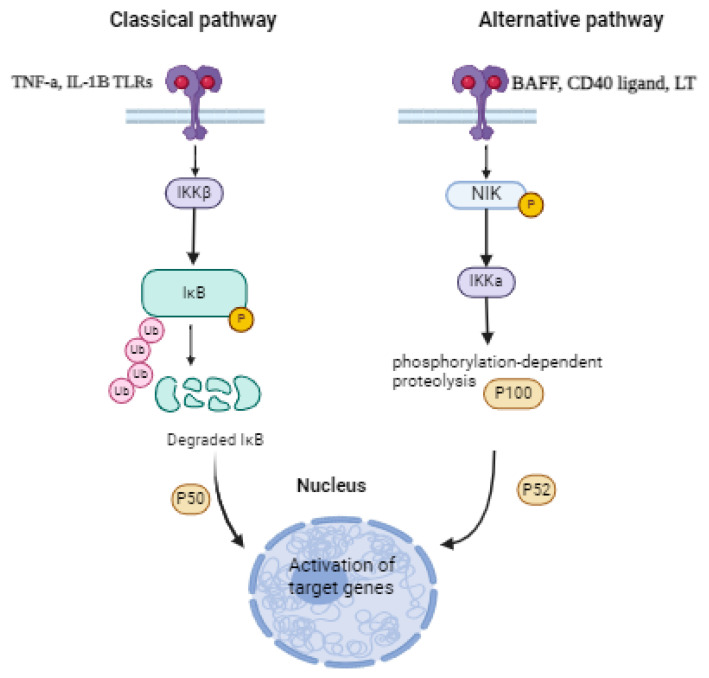
The classic and alternative pathways in activated NF-kB. The classical pathway is activated by stimuli like TNF-α, IL-1βB, and TLRs, leading to IκB degradation and the nuclear translocation of p50. In contrast, the alternative pathway, triggered by signals such as BAFF, CD40 ligand, and LT, involves NIK and IKKα to process P100, resulting in the nuclear translocation of p52. Both pathways ultimately lead to the activation of target genes. TNF-α: tumor necrosis factor-alpha; IL-1β: interleukin 1 beta; TLRs: Toll-like receptors; BAFF: B-cell activating factor; CD40 ligand: cluster of differentiation 40 ligand; LT: lymphotoxin; NIK: NF-κB-inducing kinase; IKKβ: Iκ kinase beta; IKKa: Iκ kinase alpha; IkB: inhibitor of NF-κB; Ub: ubiquitin. Created with BioRender. (https://BioRender.com). Accessed on 13 May 2024.

The classical/canonical and alternative/noncanonical signaling pathways are two well-characterized types of pathways that mediate NF-kB activation. Pro-inflammatory signals or development-related factors are the primary activators of these pathways. Despite having different biological roles and signaling pathways, they engage in a complex crosstalk that controls the various ways of NF-kB functions in response to different circumstances [46]. Several immune mediators, such as pro-inflammatory cytokines (TNF-α, IL-1β), TLRs, and antigen receptor (TCR, BCR) ligation, can stimulate the classical NF-kB pathway. All of these mediators cause the IκB kinase (IKK) complex to become activated, phosphorylate IB molecules, and then degrade them through the ubiquitin–proteasome system. Once in the nucleus, the released NF-kB promotes the transcription of its target genes [19]. The alternative or noncanonical pathway, on the other hand, depends on the activation of the IKKα kinase through phosphorylation by the NF-kB-inducing kinase (NIK), which is triggered by BAFF, CD40 ligand, and Lymphotoxin (LT). IKK then triggers NF-kB precursor p100 phosphorylation-dependent proteolysis to release the mature p52 protein, which may promote the transcription of target genes (Figure 2) [47,48].

In OA, the chondrocytes change to a degradative phenotype where the NF-kB transcription factors provoke the secretion of many degradative enzymes, which include MMP1, MMP2, MMP3, MMP7, MMP8, MMP9, MMP13, and (ADAMTS) such as ADAMTS4 and ADAMTS5, resulting in articular cartilage breakdown [25,42]. Additionally, the OA chondrocytes express a wide range of NF-kB-mediated catabolic cytokines and chemokines, such as TNF-α, IL-1β, IL-6, and IL-8, which boost NF-kB activation by increasing MMPs production, reducing collagen and proteoglycan synthesis, and acting in a positive feedback loop [49]. Finally, COX2, nitric oxide synthase (NOS), and PGE2 are all induced by the NF-kB molecules, which further increase articular damage by promoting the production of catabolic factors, cartilage inflammation, and OA chondrocyte apoptosis [19,42].

### 4.2. MAPK Signaling Pathway

**Figure 3:**
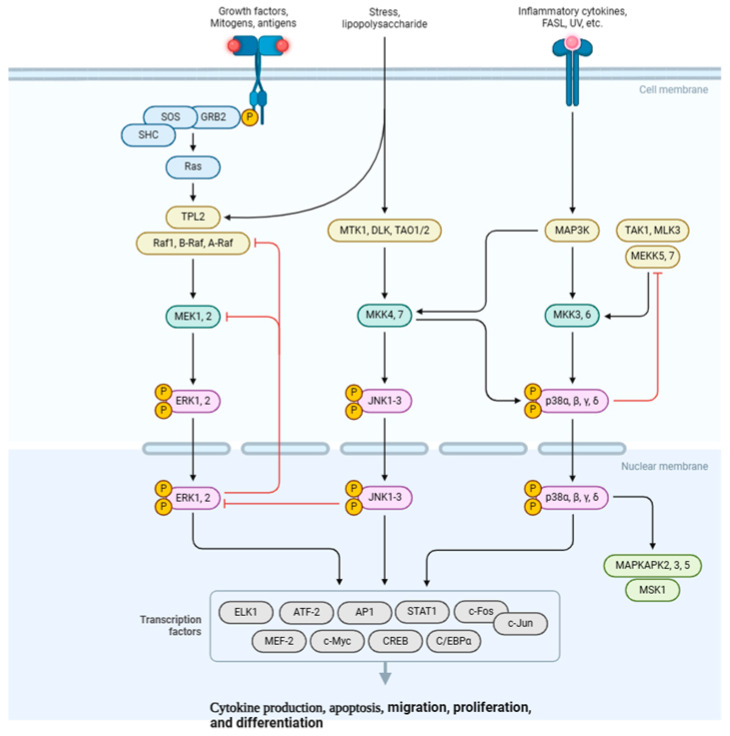
MAPK signaling pathway. This diagram illustrates the three major MAPK cascades: ERK, activated by growth factors; JNK, responsive to cellular stress; and p38, triggered by inflammatory cytokines. Each pathway involves a kinase cascade that phosphorylates and activates transcription factors in the nucleus, ultimately regulating gene expression for diverse cellular responses like cytokine production, cell growth, and death. FASL: Fas Ligand; SHC: Src Homology 2 domain containing; SOS: Son of Sevenless protein; GRB2: Growth Factor Receptor-Bound Protein 2; Ras: Rat Sarcoma protein; TPL2: Tumor Progression Locus 2; Raf1: Rapidly Accelerated Fibrosarcoma 1; MEK1, 2: Mitogen-activated protein kinase 1 and 2; ERK1, 2: Extracellular Signal-Regulated Kinase 1 and 2; MTK1: MAP Kinase 1; DLK: Dual Leucine Zipper Kinase; TAO1/2: Thousand And One Kinase 1 and 2; MKK4, 7: Mitogen-activated protein kinase 4 and 7; JNK1-3: c-Jun N-terminal Kinase 1-3; MAP3K: Mitogen-Activated Protein 3 Kinase; TAK1: Transforming growth factor-beta-activated kinase 1; MLK3: Mixed-Lineage Kinase 3; MEKK5, 7: MAP Kinase 5 and 7; MKK3, 6: Mitogen-activated protein kinase 3 and 6; MAPKAPK2, 3, 5: MAP Kinase-Activated Protein Kinase 2, 3, and 5; MSK1: Mitogen- and Stress-Activated Protein Kinase 1. Created with BioRender (https://BioRender.com). Accessed on 25 February 2025.

The mitogen-activated protein kinase (MAPK) signaling pathway is a member of the MAPK superfamily. Various external and internal stimuli, including microorganisms, extracellular signals, physical stimulation, tumor growth factor (TGF), and inflammatory cytokines, including IL-1 and IL-6, can activate it [25,50]. MAPK is a mediator that controls the downstream expression of MMPs and pro-inflammatory cytokines. It also serves as a modulator of pain. Growth factors and pro-inflammatory cytokines attach to their corresponding receptors on the cell membrane to start the process. These phosphorylate particular MAP kinases by acting as upstream activators on intracellular MAP kinases (MKKs). While MKK4 and MKK7 phosphorylate JNK1 and JNK2, MKK1 and MKK2 will activate ERK1 and ERK2, and MKK3 and MKK6. Eventually, the MKK6 will be responsible for p38 phosphorylation (Figure 3). Certain inflammatory genes, including MMPs, IL-1, and TNF-α, are upregulated as a result of activated MAP kinases activating other protein kinases and transcriptional regulatory proteins. The JNK activation can then be sustained by these cytokines, leading to increased cytokine and MMP synthesis [50,51].

In OA, the ERK, JNK, and p38 MAPK pathways are triggered in response to inflammatory stimuli, IL-1β, and TNF-α. Activation of these pathways results in the generation of inflammatory mediators, MMPs, and other catabolic factors that contribute to the breakdown of cartilage and the progression of the inflammation [52]. Moreover, MAPK signaling has a direct impact on chondrocytes. Activation of MAPK pathways can induce chondrocyte hypertrophy, death, and the upregulation of catabolic enzymes like MMPs and aggrecanases, which break down the ECM. The disparity in the degradation and synthesis of ECM is a contributing factor to the breakdown of cartilage in OA [53,54]. Another impact of MAPK signaling pathway activation is pain sensitization. MAPK signaling pathways play a role in increasing the sensitivity of pain pathways in OA. The activation of MAPK pathways in sensory neurons and glial cells located in the joint results in the secretion of pain mediators, including prostaglandins, bradykinin, and nerve growth factor (NGF). This process contributes to the severe pain that is associated with OA [55,56].

Although NF-κB and MAPK are distinct signaling pathways that become active in OA, their effects on joints frequently overlap. One notable example is their shared ability to boost the production of matrix metalloproteinases (MMPs), which are important enzymes that break down cartilage. Because of this complicated relationship, various routes can inflict identical damage while also worsening the condition. Targeting both NF-kB and MAPK pathways may improve therapy efficacy and reduce OA development [57].

### 4.3. PI3K/AKT/mTOR Signaling Pathway

**Figure 4:**
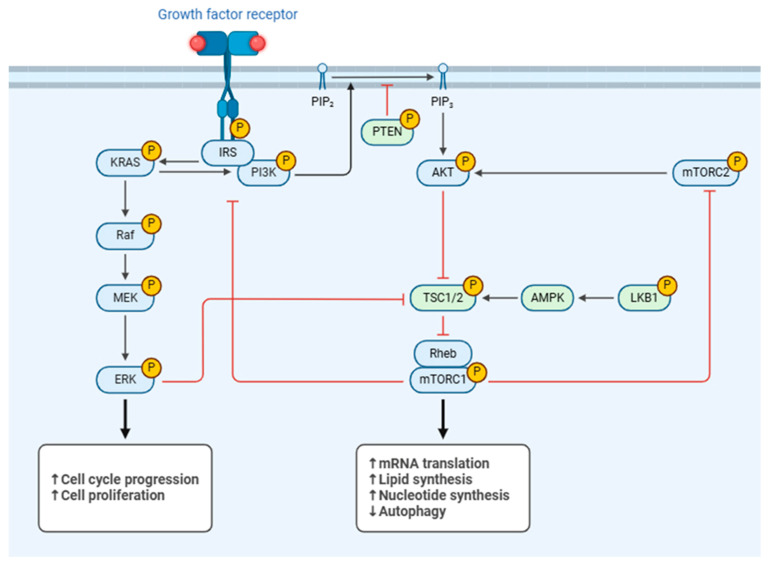
PI3K/AKT/mTOR signaling pathway. This diagram illustrates how growth factors activate the interconnected PI3K/AKT/mTOR and ERK pathways, regulating crucial cellular processes such as cell growth, proliferation, and metabolism. PIP_2_: Phosphatidylinositol 4,5-bisphosphate; PIP_3_: Phosphatidylinositol 3,4,5-trisphosphate; PTEN: Phosphatase and Tensin homolog; KRAS: Kirsten Ras; IRS: Insulin Receptor Substrate; PI3K: Phosphoinositide 3-Kinase; AKT: Protein Kinase B; mTORC: mammalian Target of Rapamycin Complex; Raf: Rapidly Accelerated Fibrosarcoma; MEK: Mitogen-activated protein kinase; ERK: Extracellular Signal-Regulated Kinase; TSC1/2: Tuberous Sclerosis Complex 1/2; AMPK: AMP-activated protein kinase; LKB1: Liver Kinase B1; Rheb: Ras Homolog Enriched in Brain; mRNA: messenger Ribonucleic Acid. ↑ increase, ↓ decrease. Created with BioRender (https://BioRender.com) accessed on 10 January 2025.

The PI3K/AKT/mTOR signaling pathway is linked to the pathophysiology of OA, and it is also crucial for preserving joint health [58]. The PI3K/AKT/mTOR pathway is a significant and complicated signaling system that involves the participation of more than 150 proteins. PI3K/AKT/mTOR plays a crucial role in maintaining cellular homeostasis by regulating several processes such as cell cycle, cell survival, inflammation, metabolism, and apoptosis through its effectors (Figure 4).

Dysregulation of these pathways is associated with the onset and progression of illnesses, like cancer, diabetes, and cardiovascular disorders. Furthermore, new research confirms the role of PI3K/AKT/mTOR in the progression of OA [58,59]. The PI3K/AKT/mTOR pathway is necessary for maintaining cartilage homeostasis [60]. Previous studies have shown that the PI3K-AKT pathway is downregulated in human cartilage tissues with OA compared to normal. OA-like chondrocytes exposed to IL-1β and TNF-α exhibit a reduction in PI3K-AKT pathway activity, also [60,61]. Several studies in OA indicate that the proteins PI3K and AKT undergo fast phosphorylation when stimulated by IL-1β [62]. Furthermore, certain biological and pharmacological substances can block the phosphorylation of PI3K, AKT, and NF-κB, as well as suppress inflammatory responses caused by IL-1β [58,63]. This suggests that the PI3K/AKT/NF-kB pathway may play a role in triggering an inflammatory response. Protein kinase A (PKA) and AKT can activate NF-kB by influencing the IkB kinases that are located upstream. Therefore, the activation of PI3K and AKT has a role in phosphorylating NF-kB p65 and facilitating its movement into the nucleus, as mentioned earlier, which in turn promotes the synthesis of inflammatory mediators [58].

### 4.4. JAK/STAT Signaling Pathway

**Figure 5:**
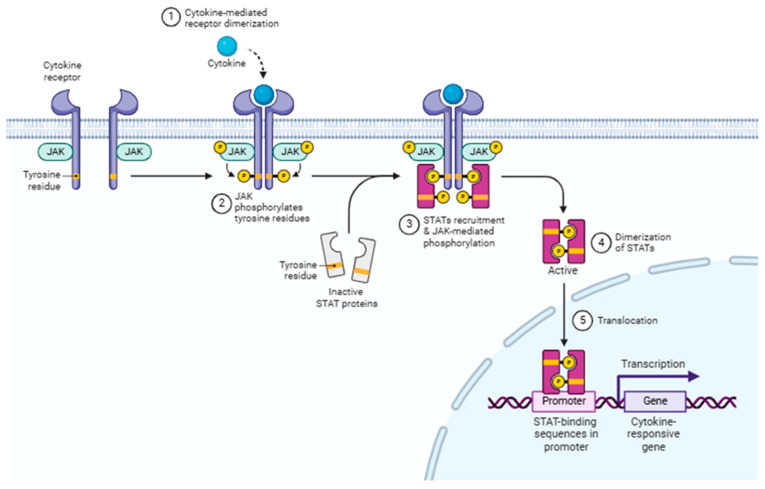
JAK/STAT signaling pathway. Cytokine binding activates JAK kinases, leading to the phosphorylation and dimerization of STAT proteins. These dimerized STATs then translocate to the nucleus to initiate the transcription of cytokine-responsive genes. JAK: Janus Kinase; STAT: Signal Transducer and Activator of Transcription. Created with BioRender (https://BioRender.com) accessed on 3 July 2024.

The Janus kinase (JAK)/signal transducer and activator of transcription (STAT) pathway is an evolutionarily conserved signaling mechanism that can be activated by a wide range of cytokines, interferons, growth factors, colony-stimulating factors, hormones, and other related molecules [64]. Tyrosine kinase-associated receptors are situated in the cell membrane, designed to interact specifically with cytokines or growth factors, subsequently leading to the activation of JAK through the phosphorylation of tyrosine residues (Figure 5) [65]. This signaling pathway is capable of facilitating signal transduction from extracellular factors to the nucleus [66]. The involvement of the JAK/STAT signaling pathway in various critical physiological activities, including cell proliferation, differentiation, immune regulation, and apoptosis, has been well established [67].

In OA, the JAK/STAT pathway is crucial in mediating the effects of pro-inflammatory cytokines on cartilage breakdown and synovial inflammation [68]. Activation of the JAK/STAT signaling pathway facilitates cartilage degradation by enhancing the expression of matrix-degrading enzymes, including MMPs and ADAMTS. It additionally stimulates the synthesis of pro-inflammatory mediators, such as NO and PGE2, which further aggravate cartilage deterioration [69]. In the synovium, the activation of the JAK/STAT pathway by cytokines such as IL-6 and IL-1β induces the proliferation of synovial fibroblasts and the synthesis of more inflammatory cytokines. This establishes a positive feedback loop that sustains inflammation and joint degradation [70].

Table 1 summarizes the unique and overlapping functions of NF-κB, MAPK, PI3K/AKT/mTOR, and JAK/STAT signaling, building on the comprehensive study of inflammatory pathways in OA. It illustrates how these pathways work together to cause cartilage deterioration, synovitis, and pain by contrasting their activators, downstream catabolic consequences, and therapeutic implications. It also identifies areas that could benefit from focused interventions. The table highlights the justification for pathway-specific treatment approaches in OA management and provides a brief reference to contextualize their interactions, especially in shared processes like MMP induction.

## 5. Mechanical Stress in OA

Mechanical stress involves the physical forces exerted on joint tissues, including shear (forces that slide across the surface), compressive (forces that press down), and tensile strain (forces that stretch the tissue). These forces play a vital role in preserving joint health in typical circumstances, as they support tissue maintenance and functionality. When these forces become excessive or abnormal, they can overwhelm the joint’s adaptive mechanisms, resulting in cartilage degradation and OA [71,72,73]. Previous studies explained that excessive mechanical load affects the progression of OA by regulating cartilage degradation [74,75]. Abnormal joint loading, frequently resulting from factors like obesity, joint injury, or misalignment, disturbs the intricate equilibrium of cartilage homeostasis [74,76]. Chondrocytes react to mechanical stimuli via mechanotransduction pathways. Under physiological conditions, these pathways contribute to the preservation of cartilage integrity through the regulation of ECM synthesis and degradation. However, excessive or abnormal mechanical stress activates the IL-1β, TNF-α, NF-kB, Wnt, TGF-β, microRNAs pathways, and the oxidative stress pathway, resulting in the increased activity of catabolic enzymes, including MMPs and ADAMTS, which break down ECM components such as collagen and proteoglycans and induce the pathological progression of OA [76,77].

## 6. Mechanotransduction Signaling Pathways in OA

Mechanical stress activates several signaling pathways in chondrocytes and other joint tissues, contributing to OA progression, including the following:

### 6.1. Wnt/β-Catenin Pathway

**Figure 6:**
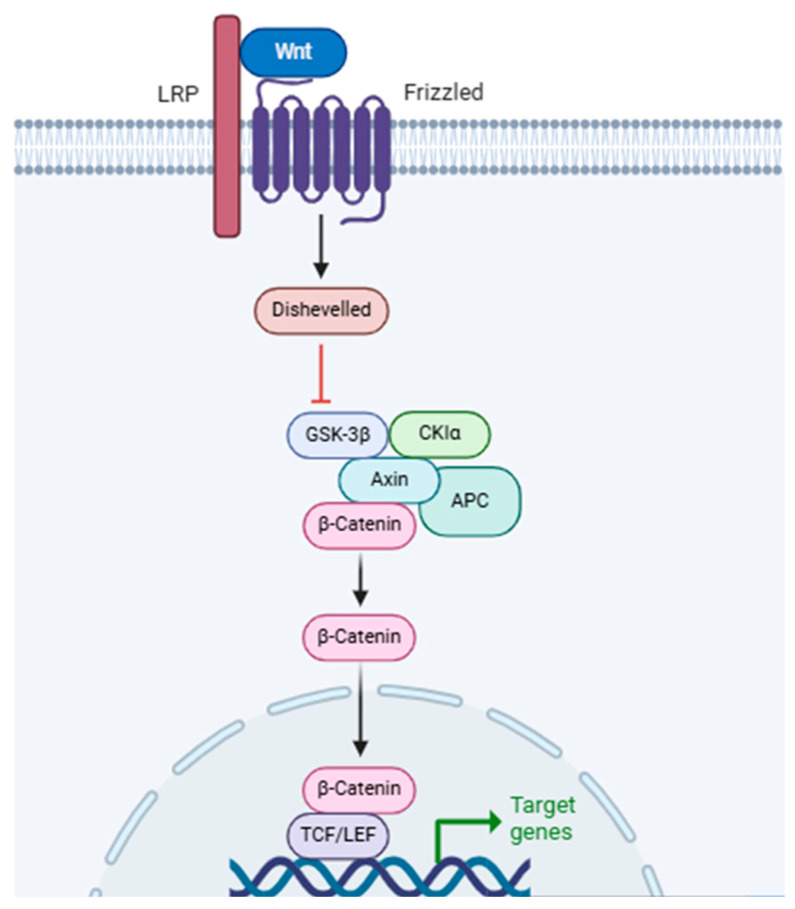
Wnt/β-catenin signaling pathway. This diagram illustrates how Wnt binding to its receptors inhibits the degradation complex, leading to β-Catenin accumulation. Accumulated β-Catenin then translocates to the nucleus to activate target gene transcription. LRP: Lipoprotein Receptor-related Protein; Wnt: Wingless-related integration site; GSK-3β: Glycogen Synthase Kinase 3 beta; CK1α: Casein Kinase 1 alpha; APC: Adenomatous Polyposis Coli; TCF/LEF: T-cell Factor/Lymphoid Enhancer Factor. Created with BioRender (https://BioRender.com). Accessed on 30 December 2024.

The Wnt/β-catenin pathway consists of a group of proteins that are essential for embryonic development and the maintenance of adult tissue homeostasis. The dysregulation of Wnt/β-catenin signaling frequently results in numerous severe illnesses [78,79]. The Wnt signaling pathways comprise both noncanonical and canonical pathways. The noncanonical Wnt pathways operate independently of β-catenin-T-cell factor/lymphoid enhancer-binding factor (TCF/LEF), including the Wnt/Ca^2+^ pathway and noncanonical Wnt planar cell polarity. The canonical Wnt pathway, or Wnt/β-catenin pathway, entails the nuclear translocation of β-catenin and the activation of target genes through TCF/LEF transcription factors (Figure 6). The canonical Wnt pathway mainly regulates cell proliferation, whereas the noncanonical Wnt pathways control cell polarity and migration; together, these pathways provide a network of reciprocal regulation.

The Wnt signaling pathway is crucial for the self-renewal of some mammalian tissues [80,81,82]. In healthy cartilage, Wnt signaling encourages the synthesis of extracellular matrix components, including collagen and proteoglycans, which are vital for the structure and function of cartilage. In OA, the dysregulation of the Wnt/β-catenin pathway can result in an imbalance between cartilage anabolism and catabolism, which contributes to the degeneration of cartilage. Furthermore, excessive Wnt/β-catenin signaling leads to abnormal chondrocyte hypertrophy and apoptosis, along with subchondral bone remodeling, which plays a role in OA pathology [83,84,85].

### 6.2. Integrin-FAK Pathway

Integrins function as transmembrane receptors, connecting the ECM with the intracellular environment [86]. Integrins, which consist of alpha and beta subunits, attach to particular components of the ECM, including collagen, fibronectin, and laminin. This interaction enables cells to detect and react to mechanical stimuli, a process referred to as mechanotransduction [87]. In chondrocytes, integrins play a crucial role in transmitting mechanical signals that influence cell proliferation and behavior, such as metabolism, survival, and matrix production [88].

**Figure 7:**
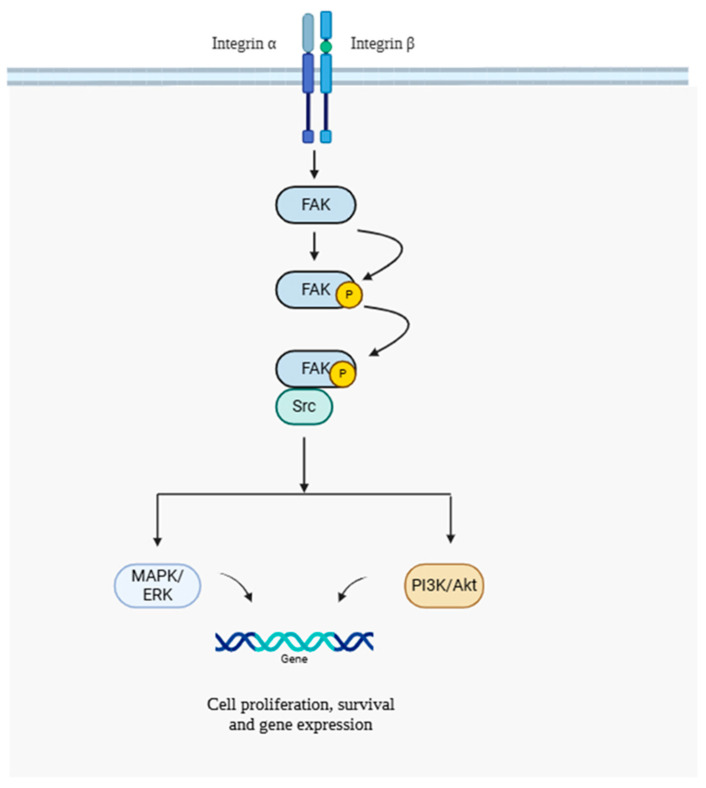
Integrin–FAK signaling pathway. This diagram illustrates how integrin activation leads to FAK phosphorylation and Src recruitment, initiating downstream signaling through MAPK/ERK and PI3K/Akt pathways to regulate cell proliferation, survival, and gene expression. FAK: Focal Adhesion Kinase; Src: Sarcoma (proto-oncogene tyrosine kinase); MAPK: Mitogen-Activated Protein Kinase; ERK: Extracellular Signal-Regulated Kinase; PI3K: Phosphoinositide 3-Kinase; Akt: Protein Kinase B. Created with BioRender (https://BioRender.com); Accessed on 15 November 2024.

Focal Adhesion Kinase (FAK) is a non-receptor tyrosine kinase that is found at focal adhesions, which are specialized structures where integrins gather and engage with the ECM [89,90]. Upon binding to their ligands, integrins experience conformational changes that lead to the activation of FAK [91]. The activation process starts with the autophosphorylation of FAK at the tyrosine residue 397 (Y397), which establishes a binding site for Src family kinases. The FAK-Src complex that forms triggers a series of downstream signaling events, leading to the activation of other pathways including MAPK/ERK, PI3K/Akt, and Rho GTPases (Figure 7). These pathways govern essential cellular functions, such as cytoskeletal reorganization, gene expression, and cell proliferation [90,91].

Within the context of OA, the Integrin–FAK pathway serves a dual purpose. Under typical circumstances, it contributes to preserving cartilage homeostasis by allowing for chondrocytes to react appropriately to mechanical stress [92,93]. In OA, this pathway becomes dysregulated, which contributes to the progression of the disease [94]. For example, irregular mechanical signaling via Integrin–FAK may result in the excessive production of matrix-degrading enzymes such as MMPs and aggrecanases, which break down the cartilage matrix [95]. Furthermore, FAK signaling has the potential to activate pro-inflammatory pathways like NF-κB, MAPK, and P13K/Akt, which can worsen joint inflammation and lead to additional cartilage damage [96].

Additionally, abnormal Integrin–FAK signaling affects the synovium [94]. Joint stiffness and fibrosis can result from synovial fibroblasts being activated by mechanical stress in OA. FAK-dependent signaling pathways that encourage fibroblast proliferation and ECM remodeling play a role in mediating this process [97]. Therefore, the Integrin–FAK pathway plays a role in the broader joint pathology associated with OA in addition to cartilage degradation.

### 6.3. Hippo–YAP/TAZ Pathway

The Hippo–YAP/TAZ pathway is a signaling network that has been preserved throughout evolution. It controls cell proliferation, differentiation, and tissue homeostasis in response to mechanical and biochemical signals [98,99]. The pathway is fundamentally made up of a kinase cascade that eventually regulates the activity of two transcriptional co-activators: Yes-associated protein (YAP) and transcriptional co-activator with PDZ-binding domain (TAZ) [98,100]. These proteins are important mediators of cellular responses to mechanical stresses, including changes in ECM stiffness, fluid shear stress, and cell shape [101,102].

**Figure 8:**
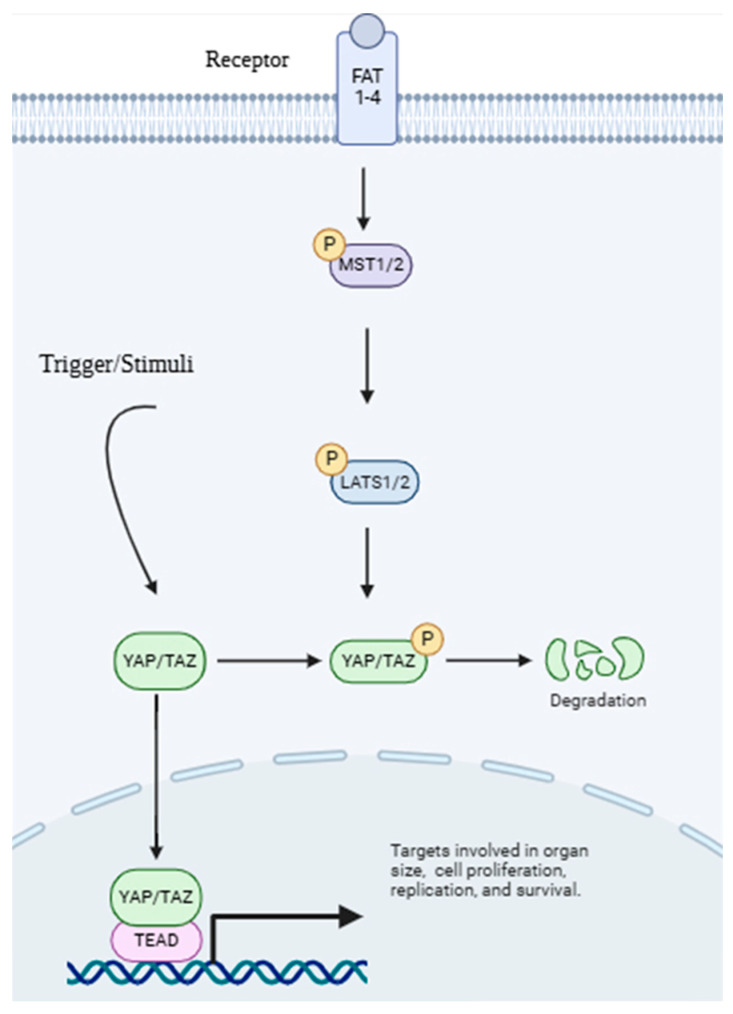
The Hippo–YAP/TAZ signaling pathway. This diagram illustrates how the Hippo pathway regulates YAP/TAZ protein activity. When active, upstream kinases phosphorylate YAP/TAZ for degradation, while its unphosphorylated form translocates to the nucleus to activate target genes involved in cell growth, proliferation, and survival. FAT1-4: FAT atypical cadherin 1-4; MST1/2: Mammalian Ste20-like protein kinase ½; LATS1/2: Large Tumor Suppressor kinase 1/2; YAP: Yes-associated protein; TAZ: Transcriptional coactivator with PDZ-binding domain; TEAD: Transcriptional Enhancer Activator Domain. Created with BioRender (https://BioRender.com) accessed on 11 September 2024.

In the classical Hippo pathway, the activation of upstream kinases (MST1/2 and LATS1/2) results in the phosphorylation and inactivation of YAP and TAZ. YAP and TAZ are kept in the cytoplasm and marked for destruction when they are phosphorylated. This stops them from entering the nucleus and controlling gene expression. However, when mechanical stimuli, such as increased ECM stiffness or mechanical loading, are present, the Hippo pathway is blocked. This translocation of YAP/TAZ to the nucleus makes them interact with transcription factors such as TEAD to activate genes that are involved in cell proliferation, survival, and ECM remodeling (Figure 8) [103,104,105].

The Hippo–YAP/TAZ pathway is essential for modulating chondrocyte reactions to mechanical stress in OA. During joint movement, chondrocytes are continuously subjected to mechanical forces. Under normal conditions, the Hippo–YAP/TAZ pathway balances anabolic and catabolic activities to maintain cartilage homeostasis. For instance, anabolic genes that encode collagen type II and aggrecan are expressed more when YAP/TAZ is activated. However, in OA, this pathway becomes dysregulated, contributing to cartilage degradation and disease progression [98].

The Hippo–YAP/TAZ pathway’s reaction to variations in ECM stiffness is one of the key mechanisms that affect OA. ECM in healthy cartilage creates a mechanically compliant environment that facilitates chondrocyte activity. However, the loss of proteoglycans and disruption of the collagen network in OA cause the ECM to become more rigid. The cartilage matrix is broken down by catabolic genes such as MMPs and ADAMTS, which are upregulated as a result of this increased stiffness, activating YAP and TAZ. Furthermore, YAP/TAZ activation encourages chondrocyte dedifferentiation, which compromises cartilage regeneration and repair by causing chondrocytes to lose their specialized phenotype and transform into a fibroblast-like condition [102,106].

The Hippo–YAP/TAZ pathway affects additional joint tissues impacted by OA in addition to cartilage. Mechanical stress in the synovium can cause synovial fibroblasts to activate YAP/TAZ, which encourages the growth of these cells and the release of inflammatory mediators such as chemokines and cytokines. This contributes to the fibrosis and inflammation of the synovium, which are characteristics of OA. Osteoblasts and osteoclasts’ YAP/TAZ activation in subchondral bone can change bone remodeling, resulting in sclerosis, and further joint dysfunction [102,107].

Overall, these mechanical pathways are not independent but crucially interact with and modulate key inflammatory signaling cascades such as NF-kB, MAPK, PI3K/AKT/mTOR, and JAK/STAT. For instance, mechanical forces, transduced through integrins or the Hippo pathway, can amplify the activation of inflammatory pathways like MAPK or NF-kB, leading to increased production of pro-inflammatory cytokines and matrix-degrading enzymes [108,109].

## 7. Interplay Between Inflammatory and Mechanical Signaling

**Figure 9:**
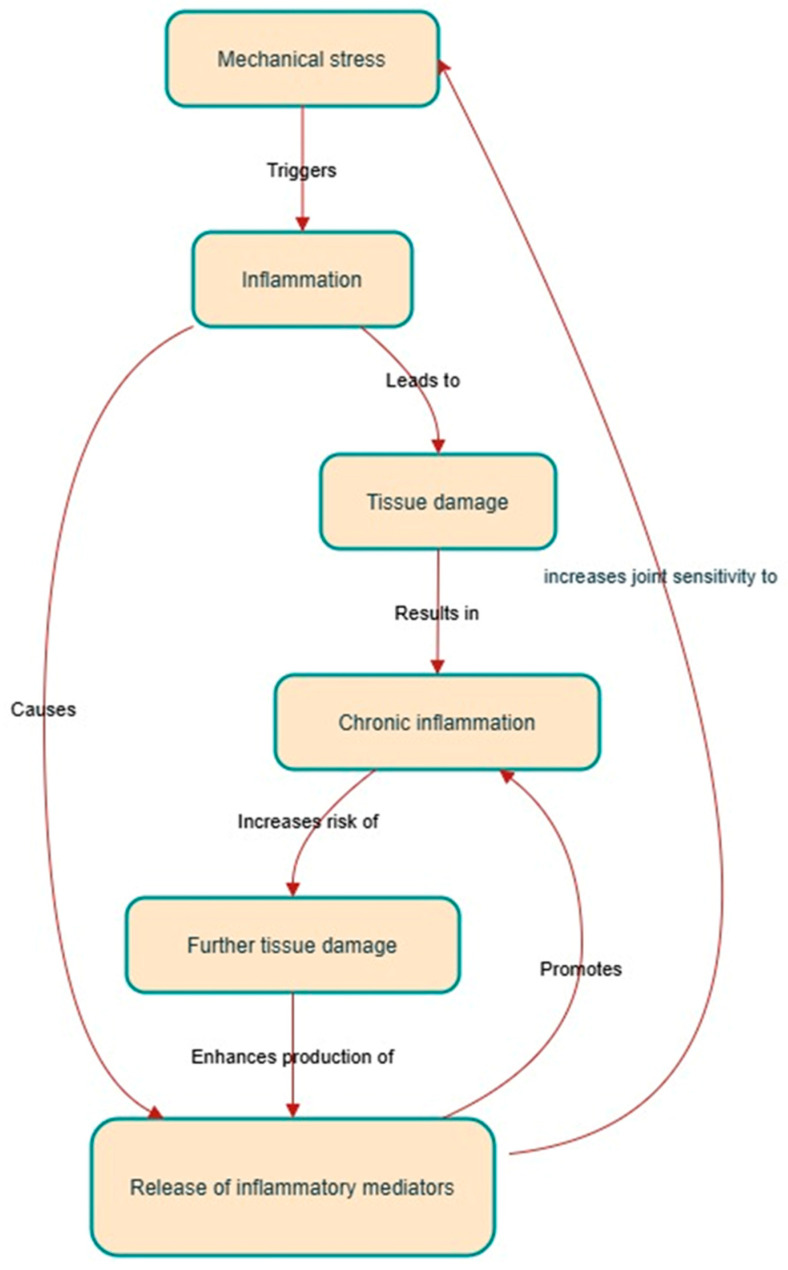
Interplay between inflammatory and mechanical signaling in OA. This diagram illustrates the vicious cycle where mechanical stress triggers inflammation, leading to tissue damage and chronic inflammation. This process is amplified by inflammatory mediators, which further increase joint sensitivity to mechanical stress and perpetuate disease progression.

Mechanical stress and inflammation had a bidirectional and reinforcing relationship. Mechanical stress, on one hand, is a known cause of inflammation through tissue damage and the activation of inflammatory pathways. Instead, inflammation makes the joint sensitive to mechanical stress by breaking down cartilage, changing tissue properties, and weakening the joint to loading [110]. This forms a vicious cycle whereby each process aggravates the other, thereby promoting the progression of OA (Figure 9) [108,111,112].

In an inflamed joint, even routine activities such as walking or climbing stairs can impose significant stress on the already compromised cartilage. This stress additionally triggers inflammatory pathways, resulting in increased cartilage destruction and synovial inflammation [76]. As time progresses, this cycle leads to the defining characteristics of OA: loss of cartilage, changes in bone structure, inflammation of the synovial membrane, and discomfort [113].

Interestingly, an in vivo study conducted by He et al. [114] showed that decreasing mechanical loading on the joint can alleviate cartilage destruction, subchondral bone changes, and inflammation in OA joints by targeting inflammatory pathways. This can help in improving daily patient activity and quality of life.

## 8. Emerging Therapeutic Strategies Targeting Inflammatory and Mechanical Pathways in OA

As previously discussed, OA is not only a single disease; it is a multifactorial condition that is characterized by cartilage degradation, synovial inflammation, and subchondral bone remodeling. Recent advances in understanding the molecular mechanisms driving OA have emphasized the importance of targeting both inflammatory and mechanical signaling pathways. This section explores emerging therapeutic strategies, including small-molecule inhibitors, biologics, regenerative medicine, and non-pharmacological approaches, that hold promise for slow disease progression and improve patient health.

### 8.1. Small-Molecule Inhibitors

Small-molecule inhibitors are molecules weighing less than 1000 Daltons and have emerged as an efficient type of treatment for OA, due to their ability to precisely target specific molecules involved in disease progression [115,116]. This specificity is a significant advantage since it attempts to maximize treatment efficacy while minimizing off-target effects and lowering the likelihood of systemic adverse effects [117]. These inhibitors can target many molecules involved in OA, including inflammatory cytokines like TNF-α and IL-1β, MMPs that cause cartilage destruction, and kinases that enhance pain and inflammation [115,118,119,120]. Small-molecule inhibitors, by carefully targeting these important key players, can disrupt the chain of events that contribute to OA progression [116]

Small-molecule inhibitors function through a variety of methods. Some inhibitors work by directly inhibiting the active site of an enzyme, preventing it from performing its activity. MMP inhibitors, for example, work by inhibiting the activity of these enzymes, reducing cartilage breakdown. Other inhibitors may interfere with intracellular signaling pathways, preventing the transmission of signals that cause inflammation and discomfort [121]. The ability to control individual molecular processes provides a more targeted approach than traditional medications.

Small-molecule inhibitors provide different advantages that make them ideal therapeutic options. Many can be given orally, which improves patient compliance in comparison to injectable therapy [122]. Furthermore, they are less expensive to produce, thereby making them more accessible to a larger patient population [123]. Perhaps most crucially, by targeting key pathways implicated in OA development, these inhibitors have the potential to alter disease progression rather than simply treating symptoms [121]. Although challenges remain, especially in minimizing the interaction with other non-target molecules, small-molecule inhibitors represent a potential therapeutic strategy for OA than many current treatments that primarily focus on pain relief.

### 8.2. Biologics

Biologics, which include monoclonal antibodies and recombinant proteins, are a rapidly developing field of OA therapy [124]. Unlike small-molecule inhibitors, which primarily target internal molecules, biologics frequently target extracellular signaling molecules or cell surface receptors, providing more options for therapeutic interventions [125].

While traditional therapy has focused mostly on symptom management, the advent of biologics has opened up new possibilities for potentially changing the disease course by targeting particular molecular pathways involved in inflammation and tissue repair [126,127,128].

One of the main mechanisms biologics work in OA is by controlling inflammation. Chronic inflammation plays an important role in the pathophysiology of OA, contributing to cartilage destruction and discomfort. Biologics can target inflammatory mediators, including IL-1β and TNF-α, which are involved in the beginning and the progression of OA [129,130]. Biologics can help reduce inflammation within the joint by neutralizing pro-inflammatory cytokines, potentially reducing the damaging processes that contribute to cartilage degradation [129,131].

Beyond that, certain biologics show potential in encouraging tissue repair and regeneration in the OA joint [132]. Growth factors, including TGF-β, IGF, and BMPs, are essential for cartilage repair and homeostasis. Biologics that provide or stimulate the production of these growth factors could help in chondrocyte proliferation and matrix formation, potentially resulting in cartilage regeneration or repair [133]. This regeneration potential distinguishes biologics from conventional medicines, which largely focus on symptom relief [134].

However, obstacles persist. A major obstacle is delivering biologics to the target tissue. Because of their size and complexity, biologics are frequently supplied via injection directly into the affected joint, which can be painful and may not always result in optimal drug concentrations at the target site. Another challenge is the possibility of immunogenicity, as biologics can occasionally cause an immunological response in the patient, resulting in severe effects. Furthermore, the long-term efficacy and safety of biologics in OA treatment must be thoroughly tested in clinical trials to assess their full potential [135].

While concerns are present, continued research and clinical trials are critical for developing and refining these techniques. Finally, biologics promise to provide more effective and targeted therapy for patients living with OA, perhaps leading to enhanced joint function and quality of life.

### 8.3. Regenerative Medicine

Regenerative medicine includes a variety of innovative methods for stimulating the body’s natural healing systems and regenerating damaged tissues [136]. In the context of OA, these methods are aimed at healing or replacing damaged articular cartilage, which is the smooth, protective tissue that covers the ends of bones in the joint [137,138]. The goal is not just to relieve pain, but also to restore structural integrity and functionality to the damaged joint [139]. These innovative strategies include cell-based therapies, tissue engineering, and gene therapy.

#### 8.3.1. Cell-Based Therapies

These procedures involve transplanting cells into the joint that has been damaged to encourage cartilage regeneration [140]. Mesenchymal stem cells (MSCs), derived from multiple sources, including bone marrow, adipose tissue, and umbilical cord blood, are a potential cell type due to their multipotency and capacity to develop into chondrocytes [138,141,142,143]. Another cell-based technique that has seen some efficacy in repairing cartilage abnormalities is autologous chondrocyte implantation (ACI), which involves transplanting a patient’s own grown chondrocytes [144,145,146].

#### 8.3.2. Tissue Engineering

Tissue engineering is the use of biomaterial scaffolds in conjunction with cells and growth factors to induce cartilage regeneration [147,148,149]. Hydrogels and 3D-printed scaffolds, for example, have been designed to imitate the mechanical and biochemical properties of native cartilage, thereby providing a favorable environment for chondrocyte development and matrix synthesis [150,151,152]. Several materials, including collagen, hyaluronic acid, gelatin, and synthetic polymers, are being explored for scaffold fabrication in cartilage regeneration [153,154].

#### 8.3.3. Gene Therapy

Is the process of transferring genetic material into cells that stimulates the synthesis of therapeutic proteins, such as growth factors or anti-inflammatory compounds, within the joint [155,156,157]. This method has the potential to deliver therapeutic medicines to the affected area at a sustained level [156,158].

Overall, regenerative medicine holds the promise of fundamentally changing how OA is treated. By focusing on tissue repair and regeneration, these techniques have the potential to not only manage symptoms but also restore joint function and stop or even reverse disease progression [159]. While challenges persist, continuing research and clinical trials are opening the way for a future in which regenerative medicine plays a fundamental role in the treatment of OA, providing hope for better results and a higher quality of life for those affected by this debilitating disorder.

### 8.4. Non-Pharmacological Approaches

Beyond pharmacological and molecular targeting techniques, mechanical interventions play an important role in the overall therapy of OA by directly regulating joint loading and altering cellular mechanotransduction [160,161]. These treatments attempt to minimize negative effects on articular cartilage, encourage an anabolic environment, and eventually slow down disease development [162].

#### 8.4.1. Mechanical Unloading

Mechanical unloading refers to measures for reducing excessive or abnormal stresses expressed through an osteoarthritic joint [163]. Chronic aberrant mechanical stress is known to contribute to cartilage degradation and synovial inflammation in OA, resulting in an imbalance of anabolic and catabolic processes within chondrocytes. Mechanical unloading can relieve pain, minimize inflammatory responses, and possibly prevent further structural degradation by reducing the size of these damaging loads. Recent studies demonstrated that reducing body mass and avoiding high-impact activities (e.g., running, jumping, deep squats) significantly lowers the joint contact forces and minimizes excessive stress on joints during daily activities [164,165].

Moreover, using assistive devices such as canes, crutches, or walkers can efficiently redistribute weight and decrease the overall load on an affected joint, thus decreasing pain and facilitating mobility [166,167].

#### 8.4.2. Joint Offloading Devices

Joint offloading devices are a type of equipment or intervention (such as braces and foot orthotics) that can achieve mechanical unloading by actively dispersing forces within a joint. These devices often use external forces to transfer the load from a damaged or painful compartment to a healthier one [168,169]. Unloader braces, which are commonly used for unicompartmental knee OA, use valgus or varus forces to redistribute weight from diseased compartments (for example, medial knee OA) to healthier parts, lowering pain, improving function, and possibly slowing cartilage deterioration. Recent clinical trials have demonstrated its efficacy in pain alleviation and functional enhancement, with some comparing its cost-effectiveness to surgical treatments [168,169,170]. Foot orthotics, such as custom or wedged insoles, can also affect lower limb alignment and knee loading [171]. Emerging evidence suggests that 3D-printed orthoses significantly reduce peak knee adduction moments in medial knee OA, providing a focused biomechanical intervention [172].

#### 8.4.3. Controlled Joint Disuse

Controlled joint disuse can be defined as the controlled reduction or avoidance of harmful stress on an affected joint, especially during periods of acute pain or severe joint instability [173]. It aims to reduce pain, inflammation, and protect damaged cartilage by reducing mechanical stress on the affected joint. Recent studies display that there was reduced wear and tear, increased joint function, and faster recovery without requiring complete immobilization [161].

#### 8.4.4. Mechanotherapy

Mechanotherapy is a therapeutic method that uses precise mechanical stimulation to produce beneficial biological reactions within tissues, thereby affecting cellular mechanotransduction pathways. In the context of OA, the goal is to reactivate or repair the chondroprotective and anabolic signaling pathways that are frequently impaired in the diseased joint [72,174]. Updated research shows that proper mechanical loading can stimulate chondrocytes to produce ECM and inhibit the production of catabolic enzymes, hence enhancing cartilage integrity and slowing OA progression [72,175].

#### 8.4.5. Structured Physical Rehabilitation

Structured physical rehabilitation is a personalized, therapist-guided program that uses mechanotherapy concepts to restore joint function, reduce pain, and slow disease progression in OA. This includes strength training, which improves muscular support and load distribution; flexibility and range-of-motion exercises enable even joint loading; low-impact aerobic conditioning promotes circulation and weight control. Balance training prevents dangerous loading patterns, whilst manual treatment techniques such as joint mobilization enhance mechanics and pain management. Robotic mechanotherapy, such as continuous passive motion, has been demonstrated to improve function and reduce pain. Patient education on optimal movement and self-management promotes long-term adherence, making physical therapy a cost-effective, evidence-based treatment for OA that significantly reduces symptoms and improves mobility [176,177].

By combining these non-pharmacological mechanical therapies with molecular targets, a more comprehensive and holistic view of OA treatment can be obtained. These treatments directly target the joint’s biomechanical environment, alter cellular responses via mechanotransduction, and have the potential to significantly reduce pain, improve function, and slow down the development of cartilage deterioration.

Overall, all these emerging therapeutic strategies for OA are summarized in Table 2, categorized by their mode of intervention. Each strategy targets specific inflammatory or mechanical pathways involved in OA pathogenesis, from inhibiting cartilage-degrading enzymes (e.g., MMP-13) to modulating cytokine signaling (e.g., JAK/STAT) or promoting tissue repair (e.g., FGF-18). The table further highlights the developmental stage of these interventions, ranging from preclinical research to advanced clinical trials, offering a comprehensive overview of current innovations and their potential to reshape OA management.

## 9. Conclusions

OA is a complicated and versatile disease that extends beyond inflammation and mechanical stress. Recognizing clinical heterogeneity is critical to designing effective treatments. Future approaches will need to concentrate on personalized medication, which is informed by various molecular profiles such as inflammatory-, mechanical-, or metabolic-dominating subtypes. Identifying these distinct characteristics in patients with modern diagnostics will enable more targeted and precise therapies. Furthermore, novel immunological therapies are gaining traction, recognizing the importance of the immune system in OA pathogenesis. Researchers and clinicians can develop more effective, patient-specific strategies to manage and perhaps modify OA progression by integrating insights from molecular phenotyping and exploring novel immunomodulatory treatments, thereby improving patient outcomes.

## Figures and Tables

**Table 1:** Comparison of key inflammatory signaling pathways in OA pathogenesis.

Pathway	Key Activators	Major Downstream Effects in OA
NF-κB	IL-1β, TNF-α, TLRs, mechanical stress	-Upregulates MMPs (1, 3, 13), ADAMTS (4, 5), COX-2, NOS, PGE2. -Promotes catabolic cytokine production (IL-6, IL-8). -Induces chondrocyte apoptosis.
MAPK	IL-1β, TNF-α, mechanical stress, TGF-β	-Activates ERK, JNK, p38 → upregulates MMPs, IL-1, TNF-α. -Induces chondrocyte hypertrophy/death. -Mediates pain sensitization (NGF, prostaglandins).
PI3K/AKT/mTOR	IL-1β, TNF-α, growth factors	-Downregulated in OA cartilage. -Crosstalk with NF-κB to amplify inflammation. -Regulates chondrocyte survival/apoptosis.
JAK/STAT	IL-6, IL-1β, interferons, growth factors	-Upregulates MMPs, ADAMTS, NO, and PGE2. -Synovial fibroblast proliferation. -Sustains inflammatory feedback loops.

**Table 2:** Shows emerging OA therapeutic strategies by intervention type.

Category	Strategy	Target/Pathway	Mechanism of Action	Example Interventions	Current Status (References)
Small-Molecule Inhibitors	MMP Inhibitors	MMP-13, ADAMTS-5	Block cartilage-degrading enzymes.	Cm-02/Ck-02	Preclinical [178]
	NF-κB Pathway Inhibitors	IKKβ, NF-κB	Suppress inflammatory gene expression.	SAR113945	Phase II trial [179]
	JAK Inhibitors	JAK1/2/3	Attenuate cytokine signaling.	Tofacitinib	Preclinical [70]
	WNT/β-Catenin Inhibitors	WNT pathway	Prevent chondrocyte hypertrophy.	Lorecivivint (SM04690)	Phase III trial [179]
Biologics	Anti-Cytokine Therapies	IL-1β, TNF-α, IL-6	Neutralize pro-inflammatory cytokines.	Canakinumab (Anti-IL-1β)	Phase II trial [180]
	Growth Factor Therapies	FGF-18, IGF-1	Stimulate cartilage repair.	Sprifermin (FGF-18)	Phase II trial [181]
Regenerative Medicine	Stem Cell Therapy	Mesenchymal stem cells (MSCs)	Promote cartilage regeneration via paracrine signaling.	Autologous MSC injections	Phase II trial [182]
	Senolytics	Senescent cells (p16, p21)	Clear senescent chondrocytes to reduce inflammation.	ABT263 + Dasatinib + Quercetin	Preclinical [183]
Non-Pharmacological	Mechanotherapy	Joint loading	Optimize biomechanics to reduce stress on cartilage.	Unloader knee braces	Clinical practice [184]
	Physical Rehabilitation	Muscle/joint function	Improve stability and load distribution through exercise.	Structured physical therapy	Clinical practice [177]
	Weight Management	Systemic metabolic factors	Reduce obesity-associated inflammation and joint load.	Diet/exercise programs	Clinical practice [164]

## Data Availability

No new data were created or analyzed in this study. Data sharing is not applicable to this article.
